# *E. coli* Secretome Metabolically Modulates MDA-MB-231 Breast Cancer Cells’ Energy Metabolism

**DOI:** 10.3390/ijms24044219

**Published:** 2023-02-20

**Authors:** Reem H. AlMalki, Rajaa Sebaa, Mysoon M. Al-Ansari, Monther Al-Alwan, Moudi A. Alwehaibi, Anas M. Abdel Rahman

**Affiliations:** 1 Department of Botany and Microbiology, College of Science, King Saud University, Riyadh 11451, Saudi Arabia; 439203044@student.ksu.edu.sa (R.H.A.); 441203139@student.ksu.edu.sa (M.A.A.); 2 Department of Medical Laboratories, College of Applied Medical Sciences, Shaqra University, Al-Dawadmi 17472, Saudi Arabia; r.sebaa@su.edu.sa; 3 Stem Cell and Tissue Re-Engineering Program, King Faisal Specialist Hospital and Research Centre (KFSHRC), Riyadh 11211, Saudi Arabia; malwan@kfshrc.edu.sa; 4 College of Medicine, Al-Faisal University, Riyadh 11533, Saudi Arabia; 5 Metabolomics Section, Department of Clinical Genomics, Center for Genomics Medicine, King Faisal Specialist Hospital and Research Centre (KFSHRC), Riyadh 11211, Saudi Arabia; 6 Department of Biochemistry and Molecular Medicine, College of Medicine, Al Faisal University, Riyadh 11533, Saudi Arabia

**Keywords:** microbiome, *E. coli* secretome, breast cancer cells, metabolites, metabolomics, high-resolution mass spectrometry

## Abstract

Breast cancer (BC) is commonly diagnosed in women. BC cells are associated with altered metabolism, which is essential to support their energetic requirements, cellular proliferation, and continuous survival. The altered metabolism of BC cells is a result of the genetic abnormalities of BC cells. Risk factors can also enhance it, including age, lifestyle, hormone disturbances, etc. Other unknown BC-promoting risk factors are under scientific investigation. One of these investigated factors is the microbiome. However, whether the breast microbiome found in the BC tissue microenvironment can impact BC cells has not been studied. We hypothesized that *E. coli*, part of a normal breast microbiome with more presence in BC tissue, secretes metabolic molecules that could alter BC cells’ metabolism to maintain their survival. Thus, we directly examined the impact of the *E. coli* secretome on the metabolism of BC cells in vitro. MDA-MB-231 cells, an in vitro model of aggressive triple-negative BC cells, were treated with the *E. coli* secretome at different time points, followed by untargeted metabolomics analyses via liquid chromatography–mass spectrometry to identify metabolic alterations in the treated BC cell lines. MDA-MB-231 cells that were not treated were used as controls. Moreover, metabolomic analyses were performed on the *E. coli* secretome to profile the most significant bacterial metabolites affecting the metabolism of the treated BC cell lines. The metabolomics results revealed about 15 metabolites that potentially have indirect roles in cancer metabolism that were secreted from *E. coli* in the culture media of MDA-MB-231 cells. The cells treated with the *E. coli* secretome showed 105 dysregulated cellular metabolites compared to controls. The dysregulated cellular metabolites were involved in the metabolism of fructose and mannose, sphingolipids, amino acids, fatty acids, amino sugar, nucleotide sugar, and pyrimidine, which are vital pathways required for the pathogenesis of BC. Our findings are the first to show that the *E. coli* secretome modulates the BC cells’ energy metabolism, highlighting insights into the possibility of altered metabolic events in BC tissue in the actual BC tissue microenvironment that are potentially induced by the local bacteria. Our study provides metabolic data that could be as a basis for future studies searching for the underlying mechanisms mediated by bacteria and their secretome to alter the metabolism of BC cells.

## 1. Introduction

Breast cancer (BC) is recognized as one of the most common types of cancer worldwide [1]. In Saudi Arabia (2018), the age-standardized rates (ASR) of BC incidence and mortality were 27.3 and 7.5 per 100,000 Saudi women, respectively (IARC, 2020) [2]. Etiologically, BC can be developed by several promoting factors categorized as genetic or non-genetic. From the genetic aspect, BC is commonly caused by mutations in nuclear genes, including BC gene 1 (BRCA1) and BC gene 2 (BRCA2), with other concerning BC-related genes [3]. On the other hand, non-genetic factors, including family history, age, menopause, hormonal disruption, diet, physical inactivity, obesity, and alcohol intake, can increase the risk of developing BC [4,5,6,7]. One of the hallmarks of BC is dysregulated cellular energy metabolism, which is required to meet the energetic requirements, induce massive proliferation, and support the continued survival of BC cells [8,9,10,11,12,13]. Examples of dysregulated metabolism are as follows: (a) upregulated glycolysis to support the increased demand for ATP [14]; (b) increased lipid and phospholipid metabolism, including de novo lipogenesis, to promote the generation of new cancer cells and their cellular membranes and organelles [15]; (c) activated mitochondrial fatty acid β-oxidation and mitochondrial biogenesis to enhance the proliferation of BC cells and to supply the needed energetic precursor for cancer growth [16,17]; (d) glutaminolysis, providing metabolites for tumor growth and invasiveness through membrane trafficking [18]; and (e) the pentose phosphate pathway and its related proteins are unregulated to generate ribose phosphate for the biosynthesis of nucleotides and to produce nicotinamide adenine dinucleotide phosphate for the biosynthesis of lipids and defense against reactive oxygen species [19]. The dysregulated energy metabolism of BC cells results from the genetic abnormalities of BC cells. It can be affected by intrinsic or extrinsic stimuli, including secreted metabolites (oncometabolites) from cancer cells themselves, the cancer tissue microenvironment, the extracellular matrix, and the breast microbiome (part of the breast microenvironment) [7,20,21,22]. Multiple studies that identified oncometabolites and their associations with cancer progression were extensively reviewed in [23].

Interestingly, it was recently discovered that breast tissue has a healthy local microbiome [24,25,26]. Multiple studies confirmed the existence and abundance of microbiota in BC tissue compared to healthy breast tissue [5,6,26,27]. For instance, 668 breast tissue samples collected from The Cancer Genome Atlas (TCGA) were studied. Their findings showed abundant *Mycobacterium fortuitum* and *Mycobacterium phlei* in BC tissue compared to the normal adjacent tissue [28]. Another study investigated the abundance of microbiomes in BC tissue compared to normal adjacent breast tissue, finding relatively increased abundances of *Methylobacterium radiotolerans* in BC tissue and *Sphingomonas yanoikuyae* in normal adjacent tissue [4].

Moreover, *E. coli* was found to be abundant in the breast cancer tissue microenvironment compared to healthy women without specific strain characterization [7]. Few studies have investigated the composition and occurrence of microbiomes in BC and normal breast tissue by distinguishing the metabolic differences between BC and normal breast tissue [8,29]. Considering these findings, it is suggested that there are potential roles of the bacterial microbiome and their secreted metabolites in BC pathogenesis; however, these potential roles have not been directly examined. Indeed, the complex interplay of BC, the local microbiome secretome, and altered metabolism merit investigation.

Metabolomics, a study of small metabolites, has been used to understand diseases’ pathogenesis, including BC, from different aspects [30]. Metabolomics is a promising approach to identifying and quantifying the endogenous and exogenous small molecules in a biological system, such as carbohydrates, nucleic acids, amino acids, and lipids [31,32]. Metabolomics identifies possible metabolic biomarkers associated with a disease phenotype (metabotype) [33,34,35,36,37]. Metabolomics can be targeted or untargeted analyses using nuclear magnetic resonance (NMR) or mass spectrometry (MS). MS is frequently used alongside separation techniques such as liquid or gas chromatography. This approach might be used in various biological samples, including tissue, plasma, urine, and conditioned culture media. Metabolomics relies on the use of advanced analytical instruments in bioinformatics [38,39]. Since metabolomics has increasingly gained interest for studying different diseases, we aimed to utilize an untargeted metabolomics approach to examine the metabolic impacts of the secretome of *E. coli*, a representative local bacterium found in BC tissues [29], on the metabolism of MDA-MB-231 cells, an in vitro model of aggressive triple-negative BC, to have a better understanding of the metabolic connection between the microbiome and BC in the BC microenvironment.

## 2. Results

### 2.1. Effect of E. coli Secretome on MDA-MB-231 Cells

**Figure 1:**
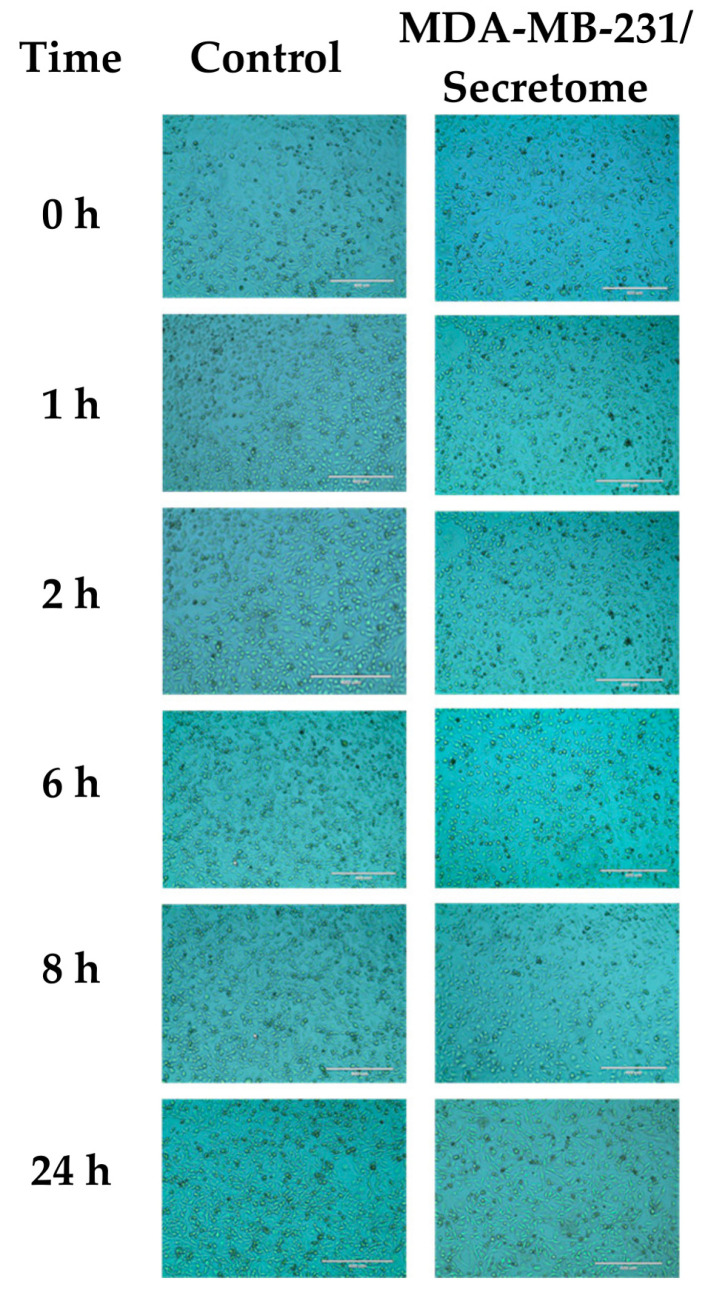
Morphological changes in MDA-MB-231 cells treated with 10% *E. coli* secretome at different time points (0, 1, 2, 6, 8, and 24 h). Magnification: 10×.

Previous studies have been providing evidence supporting the relationship between microbiota, abundance, and metabolites in BC progression and development [5,7,8]. In this study, the effect of a bacteria secretome on BC cells’ metabolism was tested by using the *E. coli* secretome for a cellular treatment of the MDA-MB-231 cell line. First, the *E. coli* growth density was measured (OD = 1.5 in 1 mL/LB) to prepare the *E. coli* secretome. Therefore, MDA-MB-231 cells were treated at different time points (0, 1, 2, 6, 8, and 24 h) and then observed at the microscopic level. Figure 1 shows no significant changes in the cellular phenotype of the treated cells compared to controls at all tested time points.

### 2.2. Metabolites of E. coli Secretome

**Figure 2:**
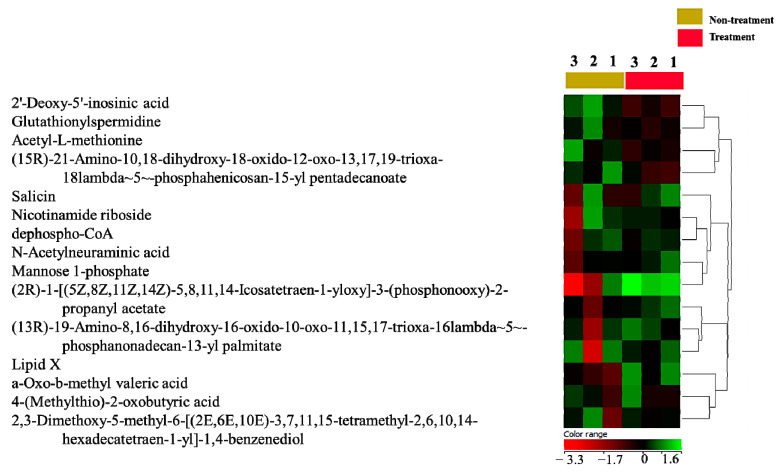
*E. coli* secretome metabolite identification. A heatmap and hierarchical cluster analysis showed only 15 excreted *E. coli* metabolites in the culture media of MDA-MB-231 cells compared to the controls’ conditioned media.

Next, the *E. coli* secretome was profiled using the metabolomics platform by comparing the culture media with and without treatment at baseline. A binary comparison using a Volcano plot (no correction, *p*-value ≤ 0.05, and FC cutoff of 2) revealed 894 metabolites at baseline, of which 559 were upregulated and 335 were downregulated among the treated cells compared to control at baseline. The *E. coli* metabolome database identified 53 metabolites. A heatmap based on Pearson’s correlation coefficient and average linkage methods identified 15 metabolites that were higher at baseline, as shown in Figure 2 and Table S1.

### 2.3. Metabolomics Profile of MDA-MB-231 Cells

**Figure 3:**
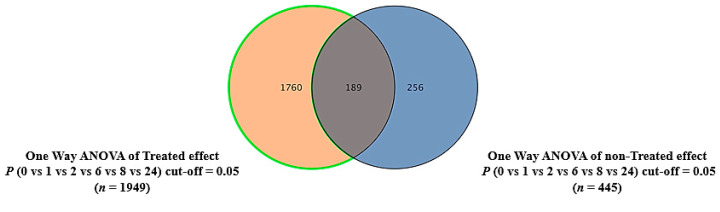
Excluded culture media backgrounds at the experimental time points. A Venn diagram showing the treated (*n* = 1949) and non-treated (*n* = 449) significant features at different time points (0, 1, 2, 6, 8, and 24 h). A group of 1760 features was consistently dysregulated 24 h post-treatment.

In total, 17,361 mass ion features were detected in intracellular extracts in both the positive and negative ionization modes. The data were deposited in MetaboLight (the accession number is MTBLS4317). After missing value exclusion and imputation, groups of 1949 and 445 metabolites were significantly dysregulated between the different time points in the treated and non-treated groups, respectively, using a one-way ANOVA (Tukey’s post hoc FDR *p* < 0.05). The culture and incubation backgrounds were excluded from the treated samples using a Venn diagram, and 1760 features remained for further analyses (Figure 3).

**Figure 4:**
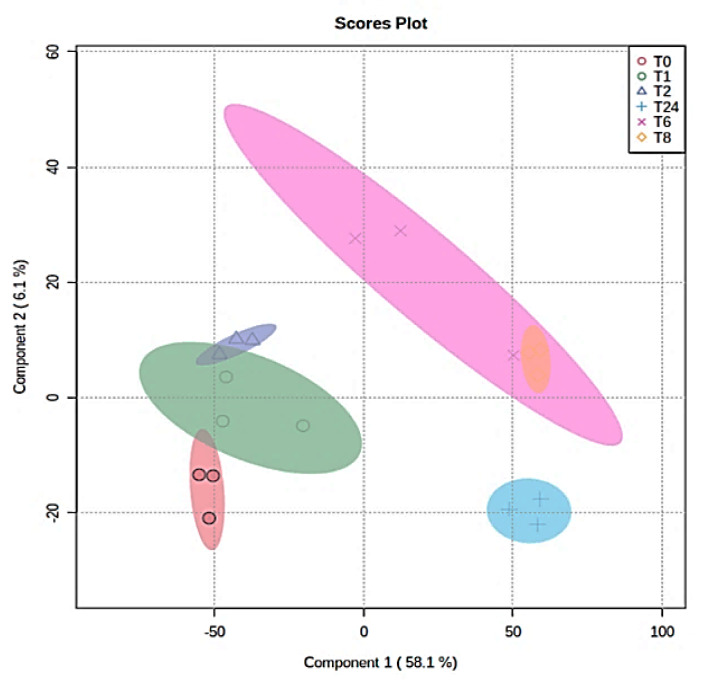
Sample clustering and group separation based on a group of 1760 features. PLS-DA for the effect of the *E. coli* secretome treatment on MDA-MB-231 cells at different time points (0, 1, 2, 6, 8, and 24). O T0, O T1, ∆ T2, X T6, ◊ T8, + T24 h.

The significant separation between the post-treatment time points was evaluated using a multivariate analysis. The PLS-DA score plot showed a significant separation between the treatment time points, as shown in Figure 4.

**Figure 5:**
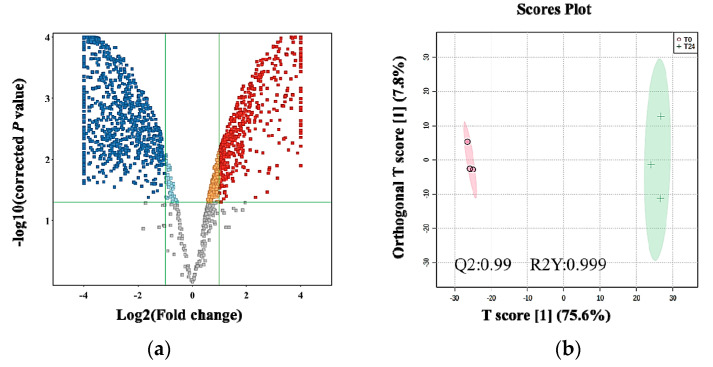
Dysregulated metabolites in MDA-MB-231 cells after 24 h of treatment with *E. coli* secretome. The culture-background-free features (*n* = 1760) were filtered using (**a**) a volcano plot, showing that 478 metabolites were upregulated (red) and 847 metabolites were downregulated (blue) after 24 h of treatment compared to the control, respectively (cutoff: FDR *p*-value ≤ 0.05 and FC 2). (**b**) An OPLS-DA model of the treatment shows a clear separation between 0 and 24 h post-treatment. The robustness of the created model was evaluated by the fitness of the model (R2Y = 0.999) and predictive ability (Q2 = 0.99) values in a larger dataset (*n* = 1000).

According to the metabolic profiling, metabolite changes were significantly detected after 24 h of treatment. A binary comparison between 24 h post-treatment was evaluated using a volcano plot (cutoff: FDR *p*-value ≤ 0.05 and FC 2). In total, 1325 metabolites were significantly dysregulated; 478 and 847 were up- and downregulated after 24 h compared to the pre-treatment sample, respectively (Figure 5a). The OPLS-DA model (Figure 5b) showed a clear separation between the two groups, with very high computed predictive ability and fitness values (Q2: 0.99 and R2Y: 0.999). Only 629 metabolites out of 1325 were identified using the HMDB and METLIN MS/MS databases. After excluding the exogenous molecules (i.e., drugs, drug metabolites, environmental exposures, etc.), 105 metabolites were retained for a further pathway analysis. In total, 53 and 52 metabolites were up- and downregulated after 24 h compared to the pre-treatment cells (Table S2).

### 2.4. The Effect of the E. coli Secretome on the Metabolites of MDA-MB-231 Cells after Treatment at Different Time Points

One hundred and five metabolites were significantly altered compared to different time points (0, 1, 2, 6, 8, and 24 h). We observed xanthosine and tetrahydrofolyl-[Glu](2) increase steadily from 1 to 8 hrs and remain constant over 24 h. At the same time, thymidine 3′,5′-cyclic monophosphate, DG (20:2/22:0/0:0), PC (22:6/22:2), and lycoperdic acid were significantly stable from baseline to 8 h and then increased significantly within 24 h. For 3,4-dihydroxyphenylglycol, 7a,12a-dihydroxy-3-oxo-4-cholenoic acid, D-threitol, 5-HEPE, LysoPE (18:0/0:0), lysoPA (O-18:0/0:0), 10,11-dihydro-20-trihydroxy-leukotriene B4, tryptophyl-cysteine, 1,2,3,4-tetrahydro-2-methyl-b-carboline, ganosporeric acid A, *N*-carbamoylputrescine, (-)-pyrifolidine, delphinidin 3-glucoside, 6b-angeloyl-3b,8b,9b-trihydroxy-7(11)-eremophilen-12,8-olide, and 2-(S-Glutathionyl)acetyl chloride, sharp increases were observed over different time points. On the other hand, 3-alpha-hydroxy-5-alpha-androstane-17-one 3-D-glucuronide, 3-phosphoadenylylselenate, Cer (d18:1/14:0), 20-trihydroxy-leukotriene-B4, glutaminyllysine, and 5-hexatriacontanone showed gradual decreases at different time points. For D-fructose 2,6-bisphosphate, inositol 1,3,4-trisphosphate, deoxyuridine triphosphate, leukotriene E3, Cer (d18:1/25:0), CDP-DG (16:0/20:4), DG (15:0/20:2/0:0), DG (15:0/24:0/0:0), PC (22:6/22:4), lysoPE (0:0/18:4), lysoPE (0:0/20:2), ganglioside GD1a (d18:1/18:1), ganglioside GT1c (d18:1/18:1), ganglioside GT2 (d18:0/14:0), ganglioside GT2 (d18:1/24:1), ganglioside GT3 (d18:0/18:0), ganglioside GT3 (d18:1/12:0), 25-methyl-1-hexacosanol, 15-hentriacontanol, asparagoside H, octadecane, 6-hydroxy-8-tricosanone, PI (20:1/PGD1), and CDP-DG (18:1-O/i-21:0), gradual decreases were observed up to 8 h and stayed the same for 24 h. All-trans-phytofluene, 4a-hydroxytetrahydrobiopterin, taurochenodeoxycholate-3-sulfate, guanidinosuccinic acid, lanthionine ketimine, galactosylceramide (d18:1/26:1), 1-(beta-D-ribofuranosyl)-1,4-dihydronicotinamide, adenosine thiamine triphosphate, bis-gamma-glutamylcysteinylbis-beta-alanine, merodesmosine, 1-acetoxy-4,6-tetradecadiene-8,10,12-triyne, hexyl heptanoate, methyl acrylate-divinylbenzene, completely hydrolyzed copolymer, and methyl 2-decenoate were stable for 8 h and then dropped off at 24 h.

### 2.5. Metabolic Pathways

**Figure 6:**
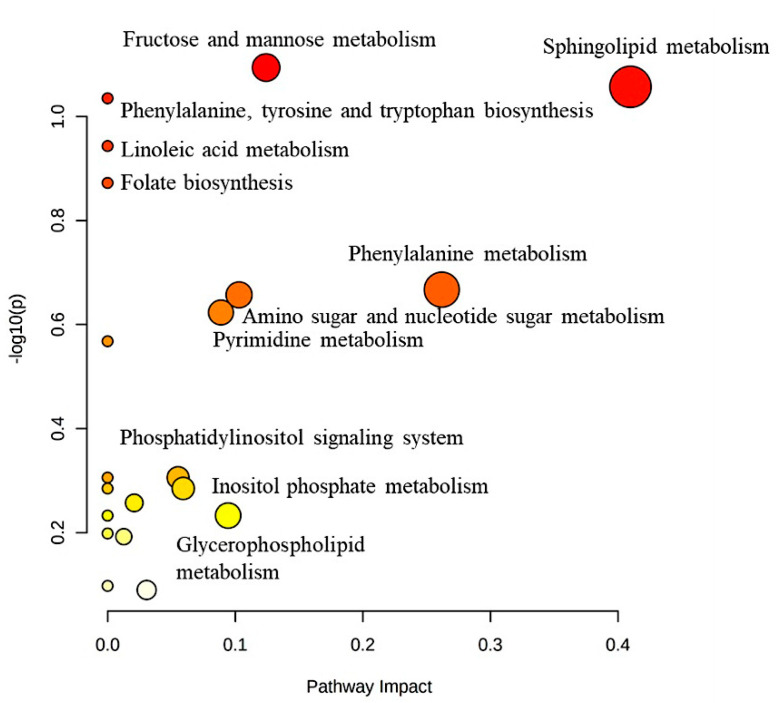
Pathway analysis for the significant metabolites dysregulated in MDA-MB-231 cells treated with the *E. coli* secretome. In total, 105 metabolites were ultimately identified as human endogenous metabolites, where 53 and 52 were up- and downregulated after 24 h of treatment with the *E. coli* secretome.

Significant metabolic pathways (*n* = 20) included lipid metabolism, such as linoleic acid metabolism, sphingolipid metabolism, glycerophospholipid metabolism, arachidonic acid metabolism, alpha-linolenic acid metabolism, and phosphatidylinositol signaling system. A total of 10 metabolites were dysregulated at 24 h post-treatment, such as 7a,12a-dihydroxy-3-oxo-4-cholenoic acid, PC (22:6)/22:2), lysoPE (18:0/0:0), lysoPA (O-18:0/0:0), and ganosporeric acid A, which were significantly upregulated. At the same time, inositol 1,3,4-trisphosphate Cer (d18:1/25:0), galactosylceramide (d18:1/26:1), lysoPE (0:0/18:4), and lysoPE (0:0/20:2) were significantly downregulated. Furthermore, four metabolites, including purine and pyrimidine biosynthesis pathways such as xanthosine and thymidine 3′,5′-cyclic monophosphate, were significantly upregulated, while deoxyuridine triphosphate and 3-phosphoadenylylselenate were significantly downregulated at 24 h post-treatment. Otherwise, the pathways for carbohydrate metabolism, including fructose and mannose metabolism, amino sugar and nucleotide sugar metabolism, and inositol phosphate metabolisms, such as D-mannose 1-phosphate and glucosamine 6-phosphate, were upregulated. At the same time, D-fructose 2,6-bisphosphate and inositol 1,3,4-trisphosphate were downregulated at 24 h post-treatment. Amino acid metabolism, including tyrosine metabolism; tryptophan metabolism; alanine, aspartate, and glutamate metabolism; and cysteine and methionine metabolism, such as 3,4-dihydroxyphenylglycol, 5′-methylthioadenosine, glucosamine 6-phosphate, and acetyl-N-formyl-5-methoxykynurenamine, were significantly upregulated at 24 h post-treatment. Folate is synthesized by all plants, fungi, bacteria, and archaea from a substrate such as tetrahydrofolyl-[Glu]. Phenylalanine metabolites, such as phenyl pyruvic acid and 4a-hydroxytetrahydrobiopterin, were up- and downregulated at 24 h post-treatment, as shown in (Figure 6).

### 2.6. Dysregulated Metabolites That Were Secreted in Culture Media after Treating MDA-MB-231 Cells with the E. coli Secretome

In total, 12,996 mass ion features were detected in extracellular extracts in both the positive and negative ionization modes. After missing value exclusion, groups of 654 and 379 metabolites were significantly dysregulated between the different time points in the treated and non-treated groups, respectively, using a one-way ANOVA (Tukey’s post hoc FDR *p* < 0.05). The culture and incubation background were excluded from the treated samples using a Venn diagram, and 579 features remained for further analyses, as displayed in Figure S1. As mentioned above (2.3), most metabolites changed in the 24 h post-treatment. Thus, we adopted a binary comparison between pre-treatment and 24 h post-treatment using a volcano plot (cutoff: FDR *p*-value ≤ 0.05 and FC 2) that revealed 347 significantly dysregulated metabolites, of which 64 and 283 metabolites were up- and downregulated at 24 h post-treatment compared to pre-treatment, respectively (Figure S2). Only 90 out of 152 metabolites were identified as endogenous, as shown in Table S3. The most affected pathways included the pentose phosphate pathway, glycolysis/gluconeogenesis, fructose and mannose metabolism, and amino sugar and nucleotide sugar metabolism (Figure S3). After comparing metabolites resulting from the data analysis of intra- and extracellular MDA-MB-231 cells treated with the *E. coli* secretome, we found 11 metabolites that represented the overlap, as shown in Table S4. The common pathways were fructose and mannose metabolism and amino sugar and nucleotide sugar metabolism.

## 3. Discussion

Although there are several leading factors involved in BC pathology, it has been suggested that the breast-tissue-associated local microbiome may contribute to BC development, growth, and progression [40]; however, the contribution of the breast-tissue-associated local microbiome to BC pathology has not been extensively studied. Thus, in this study, we explored the metabolic changes caused by the secretome of *E. coli*, a bacterium normally found in BC-tissue-associated microbiomes and in BC cells (MDA-MB-231) in vitro. Insignificantly, the *E. coli* secretome did not change the morphology of the treated MDA-MB-231 cells at the microscopic level in 24 h of treatment. However, when the treated MDA-MB-231 cells were examined at the metabolic level, the metabolic profiling revealed significant alterations in the metabolic pathways of the treated cells, including the pathways involved in the metabolism of fructose and mannose, amino acids, sphingolipids, linoleic acid, folate, nucleotide sugar, and amino sugars, pathways required for BC. Collectively, we showed that the *E. coli* secretome alters the metabolic profiling of BC cells.

### 3.1. Metabolites Released from the Secretome of E. coli May Play an Oncogenic Role

Based on our metabolic findings, 15 bacterial metabolites were significantly elevated. It could be that these 15 metabolites have potential metabolic and oncogenic impacts on BC cells. In our results of the metabolic profiling of the *E. coli* secretome, we showed that N-acetyl-L-methionine, an *E. coli* metabolite, was significantly upregulated. Accordingly, N-acetyl-L-methionine is a metabolite formed by living cells, including bacteria, and is targeted by an enzyme called aminoacylase-1, known for its high expression in tumors [41], to produce methionine and acetate [42]. It has been reported that methionine and its metabolites (polyamines) support cancer growth, as methionine is used for the formation of glutathione to scavenge reactive oxygen species that are increased in cancer cells for longer survival or for the epigenetic modification (methylation) of certain tumor-suppressor genes to downregulate their transcription for uncontrolled growth [43], suggesting a potential oncogenic role of acetyl-L-methionine. Interestingly, nicotinamide riboside was also found as one of the notably existing metabolites in the *E. coli* secretome. It has been reported that nicotinamide riboside is biosynthesized in bacteria through enzymatic reactions mediated by xanthosine phosphorylase (xapA) [44]. Nicotinamide riboside is an NAD+ precursor that can synthesize NAD+ through salvage pathways [44,45], and NAD+ is a cofactor needed for redox reactions involved in several metabolic pathways, such as glycolysis, which are highly involved in cancer pathogenesis [46]. Moreover, the N-acetylneuraminic acid detected in the *E. coli* secretome was elevated in our findings. Normally, N-acetylneuraminic acid can be found in mammalian cells and in pathogenic bacteria such as *E. coli* [47], which is consistent with our findings. N-acetylneuraminic acid is a precursor of sialic acids [48]. It has been shown that N-acetylneuraminic acid can mediate sialylation modification, which is a post-translational modification taking place on the glycan chains found in glycoproteins and glycolipids. Sialylation modification has been reported to play an important role in cancer pathology [48,49]. Furthermore, our findings showed that the *E. coli* secretome has significant levels of mannose-1-phosphate. It is known that *E. coli* biosynthesizes mannose-1-phosphate from mannose-6-phosphate via phosphomannomutase. It can be used for glycoconjugates and the glycosylation of proteins, which is another type of post-translational modification [50]. Notably, it has been reported that glycosylation and its biological impacts on the cellular proteins are associated with BC [51]. In our metabolomics profiling results of the *E. coli* secretome, glutathionylspermidine was upregulated. The production of glutathionylspermidine is biosynthesized by glutathionylspermidine synthetase/amidase in *E. coli* [52]. Previous studies showed the role of glutathionylspermidine as a glutathione provider for protein glutathionylation, which plays a fundamental role in cancer pathology and cancer resistance to chemotherapies [53,54]. In our study, we identified a bacterial metabolite known as dephospho-coenzyme A, a derivative of coenzyme A (CoA) that is involved in CoA biosynthesis and is found in eukaryotic and prokaryotic cells, including bacteria [55]. The *E. coli* secretome profile contains specific lipids and phospholipid-related metabolites, which are essential modulators for cancer cells due to their supportive roles in cancer pathologies through the membrane structure, signaling cascade, and energy substrates [56]. Collectively, these bacteria-secretome-derived metabolites may play potential oncological roles in BC cells through BC and *E. coli* crosstalk.

### 3.2. E. coli Secretome Modulates BC Cells’ Energy Metabolism

An alteration of energy metabolism is a key metabolic feature in cancer, including BC cells, which is required for tumorigenesis. In contrast to normal cells, cancer cells develop several survival and progression mechanisms, such as metabolic reprogramming. BC cells reprogram their metabolism through genetic mutations and epigenetic modifications to upregulate energy metabolism for proliferation, invasion, survival, and metastasis. Metabolic programming focuses on metabolic pathways such as glycolysis, glutaminolysis, and lipid metabolism [57]. Our study highly impacted fructose and mannose metabolism in the treated BC cell lines. These findings were consistent with other previously published studies showing the preference of BC cells for fructose as an energy substrate to provide energy and carbon atoms for the biosynthesis of nucleotides and lipid synthesis [58].

Interestingly, fructose is a preferable energy substrate for cancers in a glucose-free environment [58]. Moreover, our findings indicate that pyrimidine metabolism is affected by the *E. coli* secretome, which is in agreement with a previously reported work showing increased de novo pyrimidine synthesis in BC to support the growth of tumors by providing nucleotide precursors to eventually synthesize the macromolecules needed for new cancer cells [59]. In addition to fructose, mannose, and pyrimidine metabolism, our study showed that the treated BC cells had impacted sphingolipid metabolism compared to non-treated cells. Interestingly, it was reported that sphingolipids were upregulated in BC tissue compared to normal breast tissue [60]. Our present study provides results from the metabolic pathway analysis, showing that folate biosynthesis was targeted by the *E. coli* secretome. In line with this, it was previously shown that folate metabolism mediates one-carbon metabolism to support the growth of cancer cells through nucleotide synthesis and DNA methylation [61].

Furthermore, we showed another metabolic pathway that was affected in the treated BC cell lines: amino-acid-related metabolism. Various studies have indicated that several amino acids could induce the pathogenesis of BC. An example of those amino acids is tryptophan, which has been reported to play a cancerogenic role in BC. Using target metabolomics, human serum samples collected from patients with BC had a high level of tryptophan. It has been shown that elevated levels of tryptophan inhibit the secretion of IL-10, inducing tumorigenesis [62]. A study using positron emission tomography (PET) showed that BC tissue significantly took up a tryptophan tracer compared to the surrounding breast tissue in BC patients. Moreover, it showed that the expression of tryptophan-related enzymes and transporters was upregulated in BC tissue compared to the surrounding breast tissue [63], showing the importance of tryptophan in BC.

Moreover, using a Raman spectroscopy analysis, other aromatic amino acids, including phenylamine, tyrosine, and tryptophan, were analyzed in BC tissue and cell lines. It was revealed that the levels of these amino acids were remarkably increased in BC tissue and cell lines compared to healthy cells [64]. Our study showed that the inositol phosphate pathway was altered in treated BC cells, predicting that certain *E. coli* metabolites could modulate the metabolic pathway concerning inositol phosphate. In support of our findings, a study of the combined approaches of targeted and untargeted metabolomics on plasma samples collected from BC patients to identify BC biomarkers for detection in the early stages showed that BC in the early stages had eight altered metabolic pathways, including fatty acid, aminoacyl-tRNA biosynthesis, and inositol phosphate metabolism [65]. To sum up, our findings demonstrate that the *E. coli* secretome modulates the energy metabolism of BC cells.

Future functional studies and exploratory experiments related to the *E. coli* secretome and BC cells are required in order to understand the underlying mechanisms involved in the metabolic effects of the *E. coli* secretome on BC cells. These future studies and experiments may provide a better understanding of the metabolic effects of the local microbiome/bacteria on the BC cells in the BC tissue microenvironment and of the complexity and involvement of different risk factors in the pathology of BC. They could also assist in designing suitable therapies for BC, potentially by targeting the local pathogenic microbiome and its metabolites.

## 4. Materials and Methods

### 4.1. Bacterial Supernatant Preparation

*E. coli* ATCC 25922 bacteria (a nonpathogenic strain that is a recommended reference, making it useful for various laboratory experiments [66]) were generously donated by Abdurahman A. Niazy, Molecular and Cell Biology Laboratory (MCB Lab), College of Dentistry, King Saud University (Riyadh, Saudi Arabia). A Luria–Bertani (LB) broth medium (Sigma Aldrich (St. Louis, MO, USA)) was inoculated with *E. coli* and incubated in a shaking incubator at 100 rpm for 24 h at 37 °C. The optical density (OD) of the growth of the bacterial cell cultures was measured after 24 h at 600 nm using a spectrophotometer (Libra S22, Biochrom Ltd., Cambridge, UK). Afterward, the supernatant was collected after centrifugation at 10,000 rpm for 10 min, filtered using a 0.22 mm pore Corning disposable vacuum filtration system (Merck KGaA, Darmstadt, Germany), and stored at −80 °C for later use [67,68]. We cultured the supernatant in a DMEM/F-12 medium for 72 h to exclude any bacterial contamination.

### 4.2. Cell Culture and Treatment

MDA-MB-231 (HTB-26) cells were purchased from ATCC (Manassas, VA, USA) and cultured (1 × 10^6^ cells/mL) into plates (100 × 20 mm) using DMEM/F-12 supplemented with 10% FBS (complete medium) or 0.5% fetal bovine serum (FBS) (serum-free medium), 1% penicillin/streptomycin, and 1% L-glutamine. Then, they were incubated at 37 °C with 5% CO_2_ in a humidified chamber [69,70,71]. At 70–80% confluence, cells were treated with a 10% bacterial supernatant in SFM, while control cells were established by culturing with 10% pure LB medium in SFM. Cells were incubated at different time points (0, 1, 2, 6, 8, and 24 h) at 37 °C in 5% CO_2_ in air [72]. Next, the cell pellets and supernatant were collected for metabolite extraction. All supplements, including Dulbecco’s Modified Eagle Medium/F-12 (DMEM/F-12), FBS, penicillin/streptomycin, and L-glutamine, were obtained from Sigma (Saint Louis, MO, USA), except for the antibiotic and antimycotic solutions, which were obtained from Gibco (Grand Island, NY, USA).

### 4.3. Sample Preparation

Intracellular metabolites were extracted after removing the media and washing the cells with chilled 1x PBS. The plates were dipped in liquid nitrogen for 1 min to quench the metabolism and reduce the experimental variations. Then, 1 mL of 80% (*v*/*v*) methanol/water was added to each plate for metabolite extraction, and cells were detached using a cell scraper and transferred to Eppendorf tubes and vortex tubes in a Thermomixer (Eppendorf, AG, Germany) for 1 h at 4 °C and 600 rpm. Then, the samples were spun down for 10 min at 4 °C and 10,000 rpm (Eppendorf, SE, Germany). The supernatants were transferred to new Eppendorf tubes.

For extracellular metabolite extraction, 900 µL of 50% extraction solvent (ACN/MeOH) was added to 100 µL of medium, and the samples were vortexed in a Thermomixer (Eppendorf, AG, Germany) at 600 rpm and 4 °C for 1 h. The samples were spun down at 10,000 rpm and 4 °C for 10 min (Eppendorf, SE, Germany), and the supernatant was transferred to new Eppendorf tubes. The intra- and extracellular extracts were completely evaporated in a Speed-Vac (Christ, Germany) and then stored at −80 °C until the LC-MS analysis [73,74].

### 4.4. LC-MS Metabolomics

The dried extract samples were reconstituted in a 50% mobile phase (A: 0.1% formic acid in dH_2_O and B: 0.1% formic acid in 50% MeOH and ACN) for an LC-MS metabolomics analysis. First, 5 µL of the sample was injected into the LC column. The polar metabolites were separated in reversed-phase liquid chromatography using an ACQUITY UPLC XSelect (100 × 2.1 mm × 2.5 μm) column (Waters Ltd., Elstree, UK). The mobile phase flow rate was set at 300 μL/min, with the column temperature maintained at 55 °C and the sample manager maintained at 4 °C. Mobile phases A and B were pumped to the column in a gradient mode (0–16 min 95–5% A, 16–19 min 5% A, 19–20 min 5–95% A, and 20–22 min 95–95% A). The molecules eluted from the LC were positively or negatively ionized using an electrospray ionization source (ESI) and separated in the gas phase based on *m*/*z* using a Xevo G2-S QTOF mass spectrometer (Waters Ltd., Elstree, UK). The metabolites were ionized in the ESI source, where the source temperature was 150 °C, the desolvation temperature was 500 °C (ESI+) or 140 °C (ESI−), the capillary voltages were 3.20 kV (ESI+) or 3 kV (ESI−), the cone voltage was 40 V, the desolvation gas flow was 800.0 L/h, and the cone gas flow was 50 L/h. The collision energies of the low and high functions were set to off and 10–50 V, respectively, in the MSE data-independent acquisition (DIA) mode. The mass spectrometer was calibrated, as recommended by the vendor, with sodium formate in the range of 100–1200 Da in both ionization modes. The lock mass compound, leucine-enkephaline (an external reference to the ion *m*/*z* 556.2771 in (ESI+) and 554.2615 (ESI−)), was injected continuously, switching between the sample and the reference every 45 and 60 s for ESI+ and ESI−, respectively, for a 0.5 s scan time, a flow rate of 10 µL/min, a cone voltage of 30 V, and a collision energy of 4 V. The DIA data were collected with a Masslynx™ V4.1 workstation (Waters Inc., Milford, MA, USA) in continuum mode. QC samples were prepared with aliquots from all samples and introduced to the instrument after the randomization of each set of 10 study samples to check for system stability.

### 4.5. Data and Statistical Analyses

The raw MS data were processed following a standard pipeline, starting from alignment based on the *m*/*z* value and the ion signals’ retention time, peak picking, and signal filtering based on the peak quality using the Progenesis QI v.3.0 software from Waters (Waters Technologies, Milford, MA, USA). Features detected in at least 50% of the samples were retained for further analyses.

A multivariate statistical analysis was performed using MetaboAnalyst version 5.0 (McGill University, Montreal, QB, Canada) (http://www.metaboanalyst.ca) accessed on 3 August 2022 [75]. The imported datasets (compounds’ names and their raw abundances) were Pareto-scaled, log-transformed, and used to generate partial least squares discriminant analysis (PLS-DA) and orthogonal partial least squares discriminant analysis (OPLS-DA) models. The created OPLS-DA models were evaluated using the fitness of the model (R2Y) and predictive ability (Q2) values [76].

A univariate analysis was performed using Mass Profiler Professional v15.0 (MPP) software (Agilent, Santa Clara, CA, USA). A one-way analysis of variance (ANOVA) with Tukey’s post hoc analysis was performed among time points with significant values of less than 0.05 for the false discovery rate (FDR)-corrected p-value. A volcano plot representation was used to identify significantly altered mass features based on a fold-change (FC) cutoff of 2 and an FDR *p* < 0.05. Venn diagrams were developed using MPP v15.0 Software (Agilent Inc., Santa Clara, CA, USA), and a heatmap analysis for altered features was performed using the Pearson distance measure according to the Pearson similarity test [77].

### 4.6. Metabolite Identification

The significant features in each dataset were selected and tagged in Progenesis QI v.3.0 software from Waters (Waters Technologies, Milford, MA, USA) for peak annotation. The chemical structures of metabolites were identified by acquiring their accurate precursor masses, fragmentation patterns, and isotopic distributions in the Human Metabolome Database (HMDB) [78], METLIN MS/MS (www.metlin.scripps.edu), and the *E. coli* database to identify *E. coli* metabolites [79]. The exogenous compounds, such as drugs, food additives, and environmental compounds, were excluded from the final list.

## 5. Conclusions

Collectively, our results demonstrate for the first time that metabolomics analyses revealed that the metabolite-containing secretome from *E. coli* has possible oncogenic roles in BC cells. We propose a model wherein *E. coli*-secreted metabolites indirectly affect the metabolism of BC cells. Our metabolomics results provide a comprehensive resource for future investigations of these potential mechanisms concerning the newly discovered crosstalk of the breast microbiome within the BC microenvironment.

## Data Availability

The raw data of this study were deposited to MetaboLights and can be accessed at www.ebi.ac.uk/metabolights/MTBLS4317.

## Supplementary Materials

The following supporting information can be downloaded at https://www.mdpi.com/1422-0067/24/4/4219/s1.
