# Supplementary material for: E. coli Secretome Metabolically Modulates MDA-MB-231 Breast Cancer Cells’ Energy Metabolism
Source: Int J Mol Sci. 2023 Feb 20;24(4):4219. doi: 10.3390/ijms24044219 (PMC9964955; doi:10.3390/ijms24044219)
Supplement: Supplementary file 1 [file ijms-24-04219-s001.zip › Supplementary Figures.pdf]

Supplementary Figures.

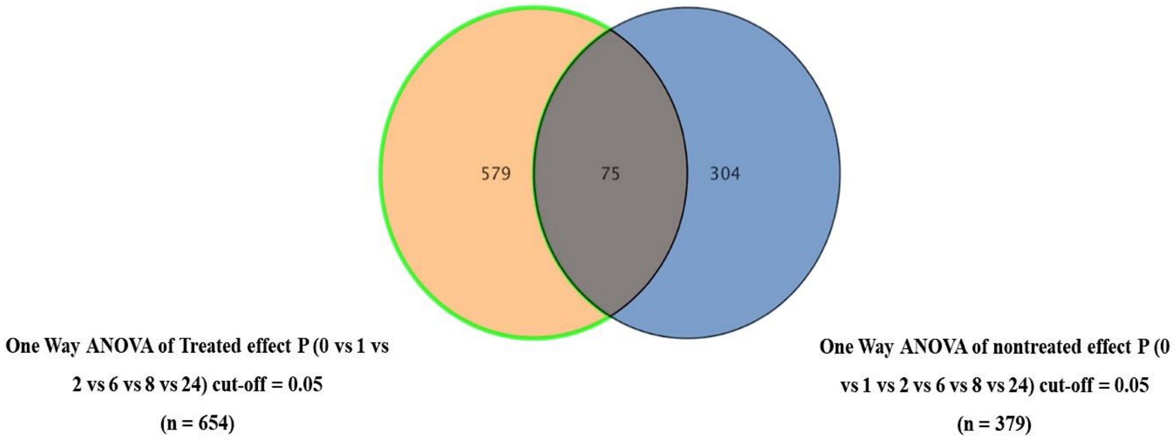

Figure S1: Excluding the background of culture media at the experimental time points. A Venn diagram showing significant features of secretion MDA-MB-231 treated (n=654) and non-treated (n=379) with *E-coli* secretome at different time points (0 ,1, 2, 6, 8 and 24 h.). A group of 579 features are consistently dysregulated in 24 hrs. post-treatment

(a) (b)

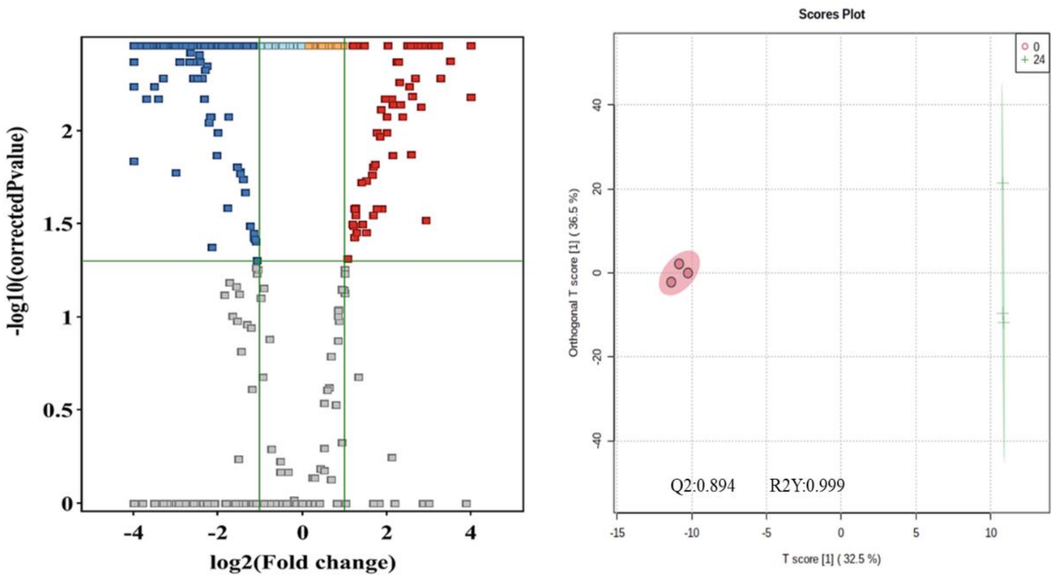

Figure S2: Dysregulated group of metabolites between MDA-MB-231 cells 0 and 24hrs post-treatment with *E-coli* secretome. The culture background-free features (n=579) were filtered using (a) A Volcano plot, where 64 (Red), and 283 (Blue) metabolites were up- and down-regulated in 24hrs. post-treatment compared to 0, respectively (Cut-off: FDR p-value ≤ 0.05, and FC 2). (b) An OPLS-DA model of treatment shows a clear separation between pre- and 24hrs. post-treatment. The robustness of the created model was evaluated by the fitness of the model (R2Y= 0.999) and predictive ability (Q2= 0.894) values in a larger dataset (n= 1000).

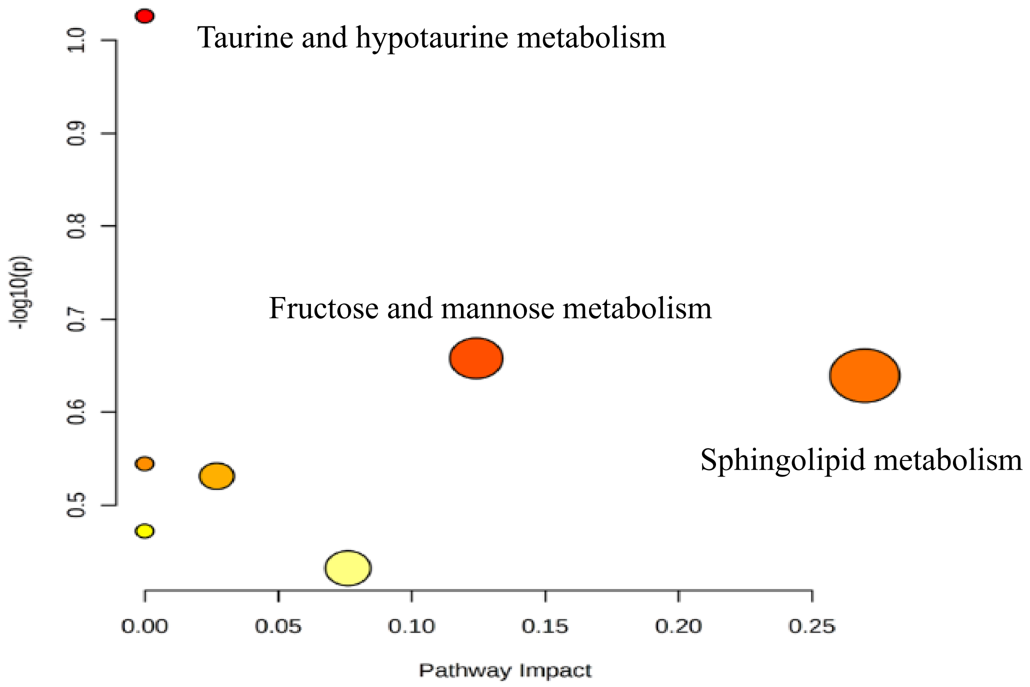

Figure S3: Pathway analysis for the significant metabolites dysregulated that secreted in culture media after treating MDA-MB-231 cells with *E. coli* secretome. 90 metabolites were ultimately identified as endogenous
